# Supplementary figures and images for: An eGFP-Col4a2 mouse model reveals basement membrane dynamics underlying hair follicle morphogenesis
Source: J Cell Biol. 2024 Dec 10;224(2):e202404003. doi: 10.1083/jcb.202404003 (PMC11629887; doi:10.1083/jcb.202404003)

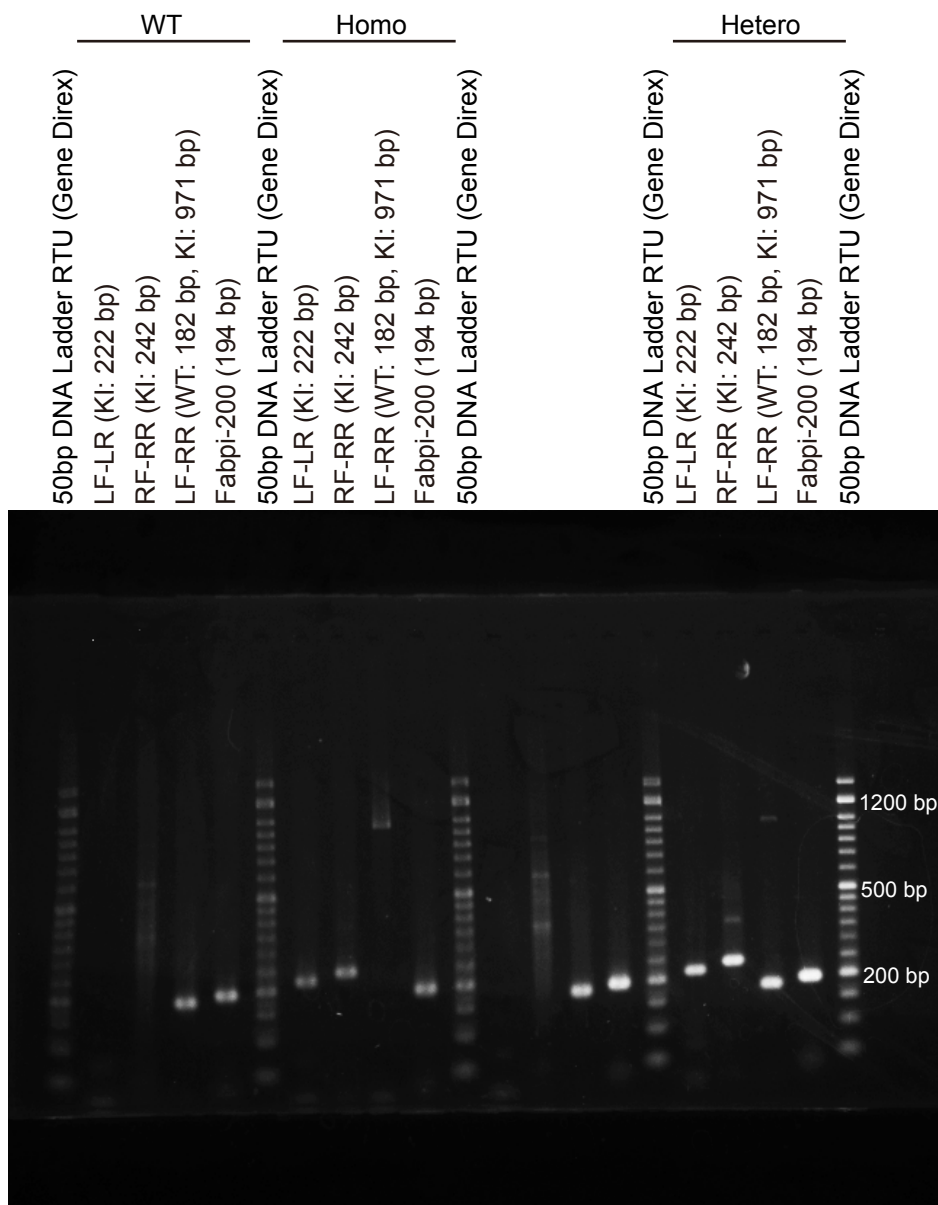

Cropped area

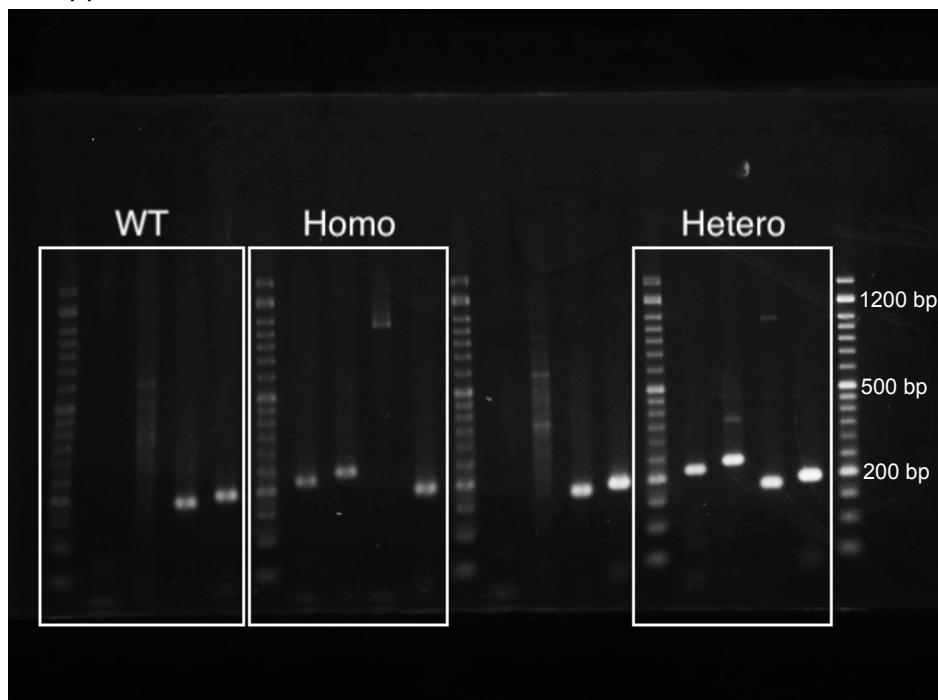

Supplement: SourceData FS1 — is the source file for Fig. S1. [file jcb_202404003_sourcedatafs1.pdf]
